# News Items

**Published:** 1880-10

**Authors:** 


### NEWS ITEMS.

CHARLES L. DANA, M. D., has been appointed to fill the chair of Comparative Physiology in the Columbia Veterinary College.

---

PLEURO-PNEUMONIA has made its appearance at Westbury and Jericho, in Queens County, and a number of cows have died from the disease in the past few days. Richard H. Robbins, of East Willerton, lost five cows last week, and others are sick. John Hubbs, of Jericho, has lost three, and others are sick. The stables infected have been quarantined. There are many dairy farms at Westbury. Yesterday a meeting of the farmers was held, and a committee was appointed to take action to prevent the spread of the disease.

---

A VIRULENT cattle disease has broken out near Champagne, Ill., from which several cows have died, and none have recovered. A post-mortem examination of one of the affected animals, showed the disease to be the veritable Texas fever. It is generally charged that the disease spread from the saw-dust bedding tramped out of the cars by Texan cattle, as the cars stood on the side tracks.

---

PROF. JAMES R. WOOD, M. D., has conferred on the professors and students of Columbia Veterinary College the privileges of his museum of natural history and comparative anatomy. This collection is arranged in Bellevue Hospital, and is one of the most elaborate and carefully selected in the country. For the purposes of instruction in the Science of Comparative Medicine, the facilities thus offered will be of inestimable value. In a future number we shall refer to this collection in greater detail.—ED.

---

CENTRAL PARK MENAGERIE.—The fallow deer doe gave birth to a healthy doe fawn, August last. The imported Kerry cow gave birth to a fine heifer calf, Sept. 1, 1880. The Cape buffalo cow gave birth to a fine bull calf Sept. 18, 1880. The above mothers and offspring are doing well.

EXCHANGES, &c., RECEIVED.—American Naturalist, Medical Record, Chicago Medical Gazette, St. Louis Clinical Record, Annals of the Anatomical and Surgical Society, Gaillard's Medical Journal, Virginia Medical Monthly, Live Stock Journal, Blacksmith and Wheelwright, Country Gentleman, Nashville Journal of Medicine and Surgery, Manchester Mirror and Farmer, Church Union, Indiana Farmer, German Agricultural Journal, Spirit of the Times, New Remedies, Medical Tribune, International Surgical Record, Schoharie Union, Daily Mirror and American, American Dairyman.

---

FOREIGN EXCHANGES:—Der Thierarzt, El Medico, Y. Cirujano Centro-Americano, Annals de Medicine Veterinäire, Thierärztliche Mittherlungen, Diario de Centro Americao.

---

PHAMPHLETS RECEIVED:—Report of the Director of the Central Park Menageeie, Statia's Hotel List and Traveller's Guide, Bulletin of the National Board of Health, Contagious Diseases of Cattle: A Prize Essay, by J. T. Duncan, V. S., Toronto, Canada.

---

DEATH OF GOLDSMITH MAID'S COLT.—Lovers of fast trotting stock will learn with regret of the death of "Consultation," at the age of one year, four months and three days, at the Fashion Stud Farm on Thursday, August 5th, 1880. It appears that the animal was placed in a field, and it is supposed that it becoming frightened dur.ng the night (possibly by some passing train), started to run, and unable to stop, struck against a picket fence. He was found at twenty minutes to five the next morning, by Superintendent E. K. Riddle, dead. Upon examination, it was found that the horse had sustained a compound fracture of the humerus, and also a rupture of a blood vessel. He was a light bay, son of Goldsmith Maid; Sire, General Washington; General Washington by General Knox, dam Lady Thorne, and had given every evidence of attaining unusual speed.

Large sums of money had been offered its owner, Mr. Smith, for the animal; but he was disinclined to part with him. Superintendent Riddle buried him where he fell.

MARK L FREY.

---

EPIZOÖTIC FEVER.—New York and vicinity is again visited with an epizoöty. It is estimated that from fifty to seventy per cent. of the animals in this city are afflicted. The disease first made its appearance in Boston, and prevailed there quite extensively Its advent in New York occurred on September 25th, since which time it has spread with great rapidity.

The disease seems to be somewhat different from the epizoöty of '72, and for want of a better term, has been styled epizoötic-entozoa. It is epidemic, and not contagious. Its development is probably due to atmospheric conditions.

In the early stages it is not severe, and as yet there are comparatively few deaths. The first symptoms are hanging of the head, shuffling gait, and a generally tired air, which are soon followed by a short, husky cough, sneezing, and a thin mucus discharge from the nostrils. The pulse is accelerated and the heart action weak. The visible mucus membranes are injected, eyes watery, and occasionally slightly pustulent secretions. As the disease advances or develops, all the symptoms increase in severity, particularly the sneezing and coughing. The temperature rises rapidly; in some cases reach as high as 107° F. The bowels become slightly affected, and the appetite lost as soon as the temperature rises. Deglutition provokes coughing. The secretion of salivary glands is somewhat increased. There is also marked tenderness over the larynx. In fact, the general symptoms are those of acute coryza and laryngetis. As yet only very mild treatment has been indicated. Purpura Hemorrhagica has made its appearance also and threatens to become epidemic.

---

An equine variola is prevalent in some sections of France. Inoculation is being tried as a remedy. The disease is reported as being much more fatal than our epizoöty.

---

A recent decision of the Westchester Supreme Court establishes the principle that a man who sells diseased cattle, not only forfeits the pay for them, but is liable in damages for the spread of the disease.

---

COLUMBIA VETERINARY COLLEGE.—The third yearly course of lectures in this excellen institution began September 29th. The introductory lecture was delivered by Prof. Louis H. Laudy, Ph D. D. V. S. Subject, The Zoöl gy and Primitive History of the Horse. As we purpose to print it in full in the next number of the ARCHIVES. we shall confine ourselves to the simple statement that the lecture was exceedingly instructive and entertaining receiving close attention and a warm reception from the audience. Prof. Bates, Dean of the College, read a report of the work done since the organization of the College. accompanied with a brief resume of the statistics of the hospital. Rev. Robt. Collyer entertained the audience with a very humorous history of his experiences as a "horse doctor;" and Prof. Heath closed the exercises with a paper on Veterinary Sanitary Science. The College is doing excelent work, and should rec eive the support and encouragement of all who are interested in the development of comparative medicine.

To quote from Prof. Bates' paper: "It is the purpose of the Faculty to so thoroughly educate the students that they shall be *competent* to treat anything in the Darwinian scale lower than man, and make the graduates the equals, if not the superiors of the human physician in every point of intelligence, skill, and knowledge of general science.

---

A SINGULAR EPIDEMIC IN CHAMPAIN COUNTY, ILL.— During the last few weeks an epidemic of sore eyes has been prevailing.

It is not very severe in its manifestations, or singular, except that it is apt to involve the optic nerve, and creates a dimness of vision in one or both eyes. It attacks both man and animals. In many respects its symptoms, in the human family, are very like those produced in hay fever. It is said to be caused by decaying rag weed (ambrosia artemisi-folia) with which old pastures, waste land, and the roadsides are overrun A case is related where an old pasture had grown up to rag-weed, which was cut, and the stock allowed to run in the field as before. It was soon observed that they were troubled with sore eyes, which, in some cases, went so far as to produce opacity of the cornea and completely blind the animals. On being turned into a fresh pasture, speedy recovery followed. Similar epidemics have made their appearance in this section before, the one of 1858 being unusually severe, leaving many of its victims with granulated lids and ulcerated corneas.—*Country Gentleman.*

